# GDF-15 (a biomarker for metformin) and the risk of COVID-19: A two-sample Mendelian randomization study

**DOI:** 10.1097/MD.0000000000034675

**Published:** 2023-09-29

**Authors:** Ya Wang, Peishan Yao, Kai Li, Shanyu Qin

**Affiliations:** a Endocrinology Department, Liuzhou Peoples’ Hospital Affiliated to Guangxi Medical University, Liuzhou, China; b Gastroenterology Department, The First Affiliated Hospital of Guangxi Medical University, Nanning, China; c Orthopedics Department, The Fourth Affiliated Hospital of Guangxi Medical University, Liuzhou, China.

**Keywords:** causal effects, COVID-19, GDF-15, Mendelian randomization, metformin

## Abstract

**Background:**

Regarding the impact of metformin on COVID-19, there are currently varying opinions from multiple studies. Growth differentiation factor 15 (GDF-15) is a biomarker of metformin use and dosage, and we used two-sample Mendelian randomization (MR) to assess the causal effect of GDF-15 (metformin) on COVID-19 susceptibility, hospitalization, and severe COVID-19, thereby guiding the selection of glucose-lowering agents for diabetic patients during the COVID-19 pandemic.

**Methods:**

Two sets of genetic tools were utilized for MR analysis, derived from publicly available genetic data. The first set was GDF-15 genome-wide association study (GWAS) data from a study with 5440 participants, while the second set was COVID-19 GWAS data from the Host Genetics Initiative (HGI) GWAS meta-analysis. The primary method used to assess causal effects was random effects inverse variance weighted estimation. Complementary methods included weighted median and MR-Egger analyses. Sensitivity analysis was performed using Cochran Q tests, MR-Egger intercept tests, MR-PRESSO, leave-one-out analyses, and funnel plots.

**Results:**

GDF-15 increased the risk of severe COVID-19 in patients (OR = 1.10, 95% CI 1.03–1.19; *P* = .006); there was no causal effect of GDF-15 on hospitalization for COVID-19 (OR = 1.02, 95% CI 0.96–1.07; *P* = .47) or susceptibility to COVID-19 in the general population (OR = 1.010, 95% CI 0.988–1.034; *P* = .354).

**Conclusions:**

Our study supports the notion that GDF-15 increases the risk of severe COVID-19 in patients. However, there is no causal relationship between GDF-15 and hospitalization or susceptibility to COVID-19.

## 1. Introduction

Coronavirus disease 2019 (COVID-19) is a viral pneumonia caused by severe acute respiratory syndrome coronavirus 2 (SARS-CoV-2), which belongs to the family of RNA viruses,^[1,2]^ since its first detection in Wuhan in November 2019, it has rapidly spread worldwide and become the most urgent public health emergency of the 21st century,^[3,4]^ according to the World Health Organization, as of June 20, 2022, there were over 535 million confirmed cases and over 6.3 million deaths worldwide.^[5]^ Due to continuous mutations in SARS-CoV-2,^[6]^ the virus may become more infectious by increasing its ability to bind with host receptors.^[6,7]^

Diabetes is a metabolic disorder characterized by various metabolic and vascular abnormalities. According to the World Health Organization, around 1.5 million people worldwide died due to diabetes in 2019, It is estimated that 537 million people are currently living with diabetes all over the world 1% and 10.5% of the adult population (20–79 years) has diabetes2. In 2019, there were 463 million people with diabetes worldwide, and it is estimated that the number of diabetic patients will increase to 700 million by 20,451.

Diabetes is a metabolic disorder characterized by various metabolic and vascular abnormalities, its incidence has been increasing yearly with the development of society and the improvement of people’s living standards, there were 463 million people with diabetes in 2019 worldwide, and it is estimated that the number of diabetic patients will increase to 700 million by 2045.^[8,9]^ Diabetic patients are at higher risk of contracting COVID-19, and their hospitalization rate, severe pneumonia, and mortality rate are higher than non-diabetic patients, as reported in many studies.^[10–16]^ The pro-inflammatory state, immune dysfunction driven by hyperglycemia,^[17,18]^ and increased microbial virulence in diabetes patients make them susceptible to virus invasion and worsen symptoms after viral infection. In addition to diabetes itself, whether hypoglycemic agents exacerbate the COVID-19 condition of diabetic patients, which is a factor that increases hospitalization, severity, and mortality rates of diabetic and COVID-19 patient?

Metformin, the first-line hypoglycemic agent for most type 2 diabetes patients, is used by approximately 150 million people worldwide.^[19]^ Several studies have reported conflicting results on the association between metformin use and COVID-19 outcomes. One study found that there was no significant difference in mortality rates between diabetic patients with COVID-19 who used metformin and those who did not use metformin, but the risk of lactic acidosis (adjusted HR, 4.71; 95% CI, 1.46–15.17; *P* = .009) and acidosis (adjusted HR, 2.50; 95% CI, 1.10–5.67; *P* = .029) was higher in the metformin group.^[20]^ Another study found that the risk of severe COVID-19 was higher among diabetic patients who used metformin compared to those who did not use metformin (OR: 3.96 [1.03–15.19], *P* = .04).^[21]^ Additionally, a retrospective analysis of 6256 diabetic patients with COVID-19 in the United States showed that the use of metformin reduced the mortality rate by 24% in female patients (OR 0.76, 95% CI, 0.60–0.96, *P* = .02), but did not affect male patients (HR 0.957, 95% CI, 0.82–1.14; *P* = .689).^[22]^ There needs to be large-scale clinical studies on whether metformin influences COVID-19, and several small observational studies have yielded inconsistent results. Observational studies are subject to confounding factors such as social culture and demographic characteristics, making it difficult to draw definitive conclusions. Mendelian randomization (MR) studies, which balance confounding factors at the genetic level, can overcome bias to a great extent.^[23,24]^ Growth differentiation factor 15 (GDF-15), also known as macrophage inhibitory cytokine-1, is a member of the transforming growth factor-beta (TGF-β) superfamily and a robust biomarker for metformin use, with its concentration reflecting the dose of metformin.^[25,26]^ In this study, we used two-sample MR analysis with single nucleotide polymorphisms (SNPs) associated with GDF-15 as instrumental variables to assess the causal relationship between GDF-15 and susceptibility, hospitalization, and severity of COVID-19. The MR approach minimizes confounding factors and reverses causation biases,^[27,28]^ revealing the causal association between metformin and susceptibility, hospitalization, and severity of COVID-19 can guide the rational use of hypoglycemic agents in diabetic patients during the pandemic and contribute to the precision treatment of diabetes.

## 2. Methods

### 2.1. Study design

**Figure 1.**
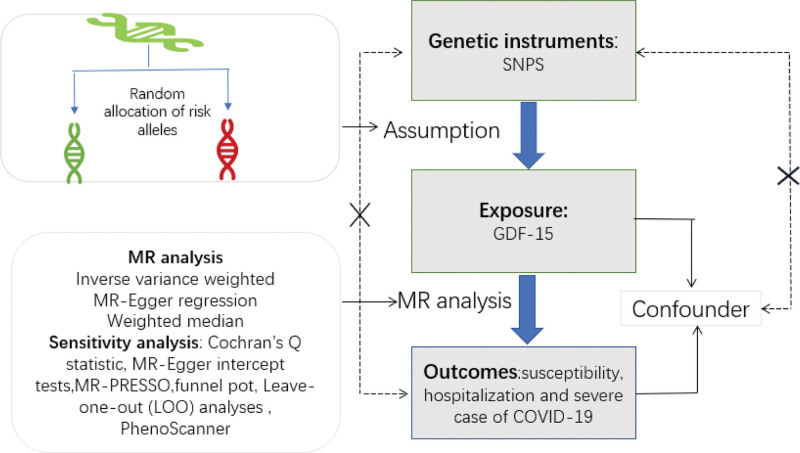
Research idea of this Mendelian randomization study to reveal the causal relationship between GDF-15 and COVID-19 susceptibility, hospitalization, and severe illness.

The general research idea of this study is shown in Figure 1, We used a two-sample MR analysis to evaluate the causal association between the metabolic marker GDF-15 of metformin and susceptibility, hospitalization, and severity of COVID-19 using large-scale genome-wide association study (GWAS) summary statistics. The offspring’s single nucleotide polymorphisms (SNPs) are randomly distributed, the Mendelian randomization (MR) analysis employs SNPs as instrumental variables, mimicking randomized controlled trials. The MR approach is based on 3 assumptions: (1) the instrumental variable is strongly associated with GDF-15; (2) the instrumental variable is independent of confounding factors; (3) the instrumental variable affects the outcome only through the risk factor we are interested in.

### 2.2. Genetic variation related to growth differentiation factor 15 (GDF-15)

Genetic prediction data for growth differentiation factor 15 (GDF-15) was sourced from a study by Au Yeung SL et al (PMID: 31161347). This research extracted genetic variations from a whole-genome association analysis of 5440 samples of European descent and adjusted the data for factors such as age, gender, systolic blood pressure, use of antihypertensive drugs, smoking status, diabetes, and year of data collection. A total of 9 genetic variations associated with GDF-15 were identified (see Table S1, Supplemental Digital Content, http://links.lww.com/MD/J980) in this research, explaining 21.5% of the variation in GDF-15. To improve the accuracy of instrumental variables, we carefully selected SNPs that meet the criteria: (1) *P* < 5 × 10^−8^; (2) using the PLINK algorithm to clump SNPs (r2 threshold ≤ 0.5 and window size ≦ 100 kb); (3) removal of SNPs that may have potential polymorphic effects, this method has been widely used in previous Mendelian studies,^[29–31]^ then we identified 4 genetic variations associated with GDF-15 (see Table S2, Supplemental Digital Content, http://links.lww.com/MD/J982), These 4 SNPs explained 8.3% of the variation in GDF-15, with an *F*-statistic of 123.3. We also conducted harmonizing processes to exclude ambiguous and palindromic SNPs.

### 2.3. COVID-19 GWAS data source

The COVID-19 GWAS data used in this study were sourced from the COVID-19 Host Genetics Initiative (HGI) GWAS meta-analyses.^[32]^ COVID-19 was categorized into 3 phenotypes: susceptibility, hospitalization, and severe COVID-19. Individuals of European descent were selected for all 3 phenotypes. The instrumental variable for susceptibility was obtained by comparing COVID-19 patients and non-COVID-19 patients (122,616 cases; 2,475,240 controls). COVID-19 patients were defined as laboratory-confirmed, clinically diagnosed, or self-reported as having COVID-19. The instrumental variable for hospitalization was obtained by comparing hospitalized COVID-19 patients to non-hospitalized COVID-19 patients or individuals not diagnosed with COVID-19 (32,519 cases; 2,062,805 controls). Hospitalization was defined as being laboratory-confirmed with COVID-19 and admitted to a hospital. Finally, the instrumental variable for severe COVID-19 was obtained by comparing severe COVID-19 patients to non-severe COVID-19 patients or individuals who were not diagnosed with COVID-19 (13,769 cases; 1,072,442 controls). Severe COVID-19 was defined as those requiring mechanical ventilation, noninvasive ventilation, or death due to COVID-19.

### 2.4. Risk factor analysis

To explore the potential mechanism linking GDF-15 to severe COVID-19 at the genetic level, we used MR to evaluate the effect of GDF-15 on common risk factors for severe COVID-19, including asthma, cerebrovascular disease, chronic kidney disease, interstitial lung disease, COPD, liver cirrhosis, diabetes, high blood glucose levels, heart failure, coronary artery disease, HIV, obesity, smoking, tuberculosis, lung cancer, and breast cancer.^[33,34]^ GWAS data for these risk factors were sourced from IEU Open GWAS project (detailed information are provided in Table 1); GDF-15 was used as the exposure, while the risk factors were used as outcomes for MR analysis.

**Table 1:** Detailed information on the GWAS for risk factors included in the analysis.

Phenotype	Participants	Consortium	Web source
Asthma	463,010	MRC-IEU	IEU
CD	218,792	NA	IEU
CKD	216,742	NA	IEU
ILD	198,955	NA	IEU
COPD	361,194	Neale lab	IEU
Cirrhosis	218,792	NA	IEU
Diabetes	461,578	MRC-IEU	IEU
Heart failure	977,323	NA	IEU
CAD	212,453	NA	IEU
HIV	218,792	NA	IEU
Obesity	463,010	MRC-IEU	IEU
smoking	607,291	GSCAN	IEU
TB	462,933	MRC-IEU	IEU
EBGL	212,911	NA	IEU
Lung cancer	374,687	UK Biobank	IEU
breast cancer	462,933	MRC-IEU	IEU

BC = breast cancer, CAD = coronary artery disease, CD = cerebrovascular diseases, CKD = chronic kidney disease, COPD = chronic obstructive pulmonary disease, EBGL = elevated blood glucose level, GC = gastric cancer, HF = heart failure, IEU = IEU OpenGWAS project (mrcieu.ac.uk), ILD = interstitial lung disease, LC = lung cancer, TB = tuberculosis.

### 2.5. Statistical analysis

After harmonizing effect alleles for GDF-15 and COVID-19 susceptibility, hospitalization, and severe illness, we used the inverse-variance weighted (IVW) method, the weighted median method, and the MR-Egger method to assess the causal effect of GDF-15 on COVID-19 susceptibility, hospitalization, and severe COVID-19 in individuals, IVW method combined the Wald ratio estimate from each SNP to obtain an overall estimate of the effect of GDF-15 on each COVID-19 subgroup and was used as the primary method, the MR-Egger and weighted median methods were used as supplementary methods. Bonferroni correction was applied with a significance threshold of *P* < .016 (α = 0.05/3).

#### 2.5.1. Sensitivity analyses.

Using the Cochran Q test, MR-Egger intercept tests, and MR-PRESSO (*P* < .05) to assess heterogeneity and pleiotropy, we also performed a leave-one-out analysis to assess the robustness of the results, the results would be unbiased if no pleiotropy were present.^[35]^ MR-PRESSO analysis detected significant outliers and attempted to reduce pleiotropy by removing these outliers.^[36]^ Leave-one-out analysis was performed by dropping each SNP associated with the exposure and conducting an IVW analysis to evaluate whether a single SNP drove the causal relationship.

In addition, we used PhenoScanner to investigate whether these SNPs were associated with potential risk factors, If an SNP was found to be associated with a risk factor (*P* < 5 × 10^−8^), it was removed as it could cause bias.^[37]^ Palindromic SNPs, such as SNPs with ambiguous minor allele frequency >0.45 or <0.55, were excluded. The analysis was conducted using the TwoSampleMR package in R4.3.0 software version.

## 3. Results

### 3.1. Causal effects of GDF-15 on COVID-19 susceptibility, hospitalization, and severe COVID-19

**Figure 2.**
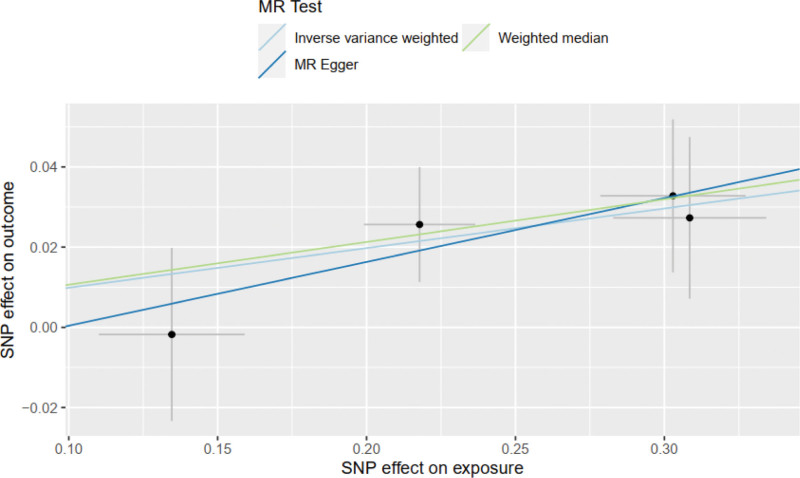
Scatter plot of severe COVID-19, which reflecting the instrumental variable increased the risk of severe COVID-19.

**Figure 3.**
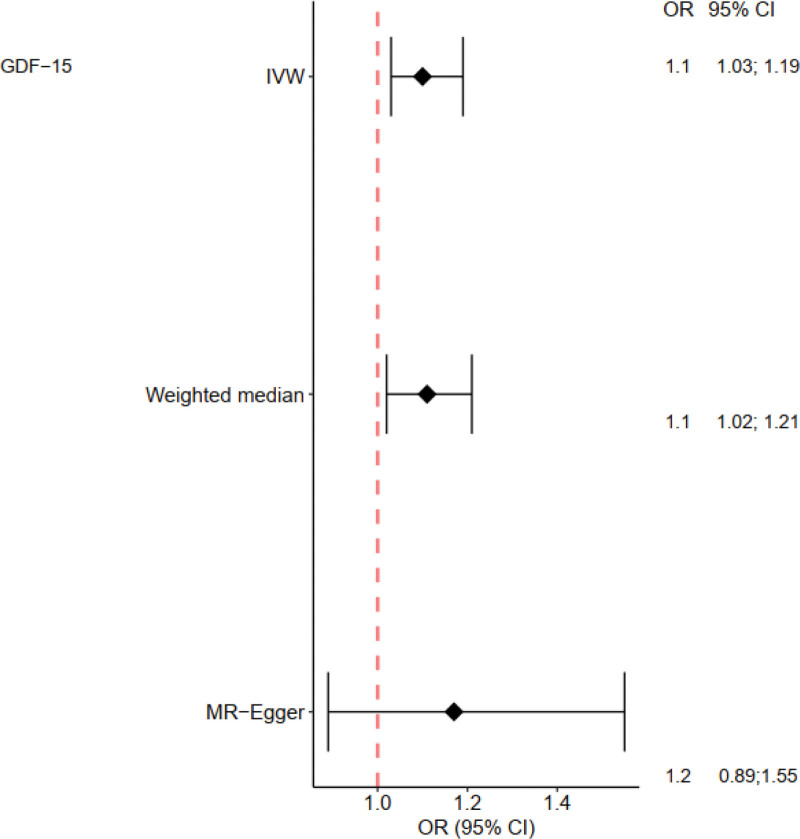
Odds ratio plot for GDF-15 and severe COVID-19. OR = odds ratio.

**Figure 4.**
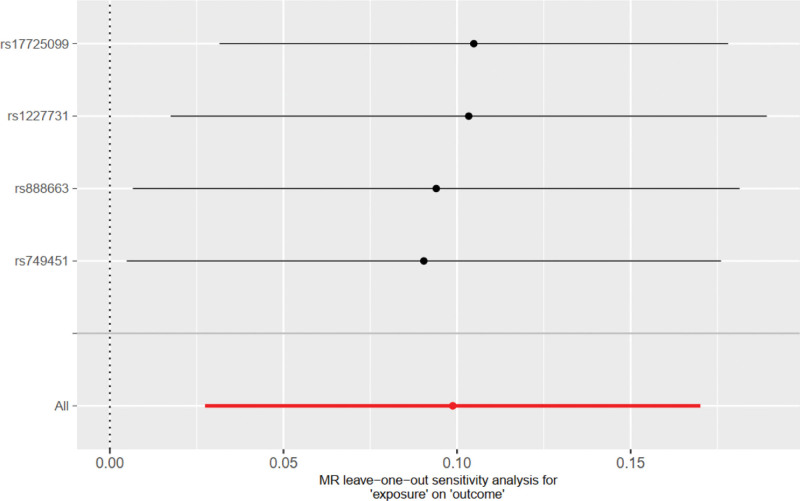
leaveoneout_plot of GDF-15 and severe COVID-19, which reflecting MR analysis results after removing SNPs one by one, which indicates the stability of the results.

The IVW method showed that GDF-15 increased the risk of severe COVID-19 in COVID patients (OR = 1.10, 95% CI 1.03–1.19; *P* = .006). The weighted median model produced similar results (OR = 1.11, 95% CI 1.02–1.21; *P* = .013). The MR-Egger model showed the same direction of effect as the above two models (OR = 1.17, 95% CI 0.89–1.55; *P* = .38) (see Figs. 2 and 3), with a power of 0.91.The Cochran Q test for heterogeneity yielded a *P*-value of .89 for IVW, and the MR-Egger intercept test yielded a *P*-value of .81, indicating no evidence of heterogeneity. Leave-one-out analysis did not detect any substantial violations of the causal effect of GDF-15 on severe COVID-19 in individuals carrying the risk alleles (see Fig. 4), indicating that the results were stable.MR-Egger regression analysis (intercept = −0.015, *P* = .71) and MR-PRESSO (*P* = .92) did not support pleiotropy. (Funnel plot of GDF-15 and severe COVID-19 see Fig. S1, Supplemental Digital Content, http://links.lww.com/MD/J983, which reflecting the SNPs of exposure are symmetrically distributed on both sides of the IVW.)

The effect of GDF-15 on hospitalization due to COVID-19 was not observed, IVW (OR = 1.02, 95% CI 0.97–1.08; *P* = .48); Weighted median (OR = 0.99, 95% CI 0.94–1.06; *P* = .91); MR-Egger (OR = 1.09, 95% CI 0.87–1.37; *P* = .52). Heterogeneity testing with Cochran Q yielded a *P*-value of .27 for IVW and .19 for MR-Egger, indicating no evidence of heterogeneity. MR-Egger regression analysis (intercept = -0.017, *P* = .59) and MR-PRESSO (*P* = .31) did not support pleiotropy. (Scatter plot, funnel plot, and leaveoneout_plot see Figs. S2–S4, Supplemental Digital Content, http://links.lww.com/MD/J984, http://links.lww.com/MD/J985, http://links.lww.com/MD/J986).

GDF-15 does not affect susceptibility to COVID-19 in the population, IVW (OR = 1.01, 95% CI 0.99–1.03; *P* = .35); weighted median (OR = 1.01, 95% CI 0.99–1.04; *P* = .34); MR-Egger (OR = 0.99, 95% CI 0.92–1.08; *P* = .95). Heterogeneity testing with Cochran Q yielded a *P*-value of 0.74 for IVW and 0.57 for MR-Egger, indicating no evidence of heterogeneity.MR-Egger regression analysis (intercept = 0.003, *P* = .76) and MR-PRESSO (*P* = .72) did not support pleiotropy. (Scatter plot, funnel plot, and leaveoneout_plot see Figs. S5–S7, Supplemental Digital Content, http://links.lww.com/MD/J987, http://links.lww.com/MD/J988, http://links.lww.com/MD/J990).

Sensitivity analysis of the causal relationship between GDF-15 and susceptibility, hospitalization, and severe cases of COVID-19 see Table 2,Causal effects of GDF-15 on common risk factors for COVID-19 see Table 3.

**Table 2:** Sensitivity analysis of the causal relationship between GDF-15 and susceptibility, hospitalization, and severe cases of COVID-19.

Exposure	Outcome	Cochran Q test		MR Egger		MR presso
		*Q* value	*P*	Intercept	*P*	*P*-value
GDF-15	Severe cases	0.61	0.89	−0.01	.70	.92
GDF-15	Hospitalization	3.93	0.26	−0.017	.599	.336
GDF-15	Susceptibility	1.247	0.741	0.003	.764	.721

**Table 3:** Causal effects of GDF-15 on common risk factors for COVID-19.

Exposure	Outcomes	IVW		Heterogeneity		MR-Egger	
		(95%CI)	*P*	*Q* valve	*P* value	Intercept	*P*
GDF-15	Asthma	0.998–0.999	.014	1.495	.473	0.00015	.719
GDF-15	CVD	0.978–1.122	.179	2.832	.418	0.028	.404
GDF-15	CKD	0.893–1.155	.805	2.057	.560	−0.052	.414
GDF-15	ILD	0.958–1.379	.133	3.107	.375	0.093	.321
GDF-15	COPD	0.999–1.001	.665	1.771	.621	0.00012	.756
GDF-15	Cirrhosis	0.909–1.299	.359	1.945	.583	0.008	.915
GDF-15	Diabetes	0.998–1.002	.508	0.445	.930	0.00058	.591
GDF-15	HF	0.943–1.027	.487	3.669	.299	0.00199	.937
GDF-15	CAD	0.947–1.032	.612	2.721	.436	0.00502	.839
GDF-15	HIV	0.651–1.481	.931	0.265	.966	−0.0722	.705
GDF-15	Obesity	0.997–0.999	.007	2.590	.459	8.999	.870
GDF-15	Smoking	0.982–1.017	.965	3.147	.369	0.00964	.321
GDF-15	TB	1.0002–1.002	.0158	3.767	.152	0.00062	.309
GDF-15	LC	0.999–1.0009	.917	0.407	.938	0.00014	.746
GDF-15	GC	0.847–1.028	.166	4.270	.233	−0.073	.18
GDF-15	BC	0.994–0.997	4.48e−07	3.601	.307	0.00063	.470
GDF-15	EBGL	0.734–1.231	0.702	0.144	.986	−0.014	.899

Abbreviations are the same as Table 1.

## 4. Discussion

Regarding the impact of metformin on COVID-19, a study reported that metformin is associated with an increased risk of disease progression in COVID-19 patients with diabetes,^[21]^ the glucose and lactate dehydrogenase levels in the group taking metformin were higher than those in the control group.^[21]^ Gao et al reported that metformin has more life-threatening complications and a higher risk of disease progression during hospitalization,^[21]^ which is consistent with the direction of our research results. However, there are also many reports that contradict our research conclusion, suggesting that metformin may have positive effects on COVID-19 by improving blood sugar, reducing weight, and improving insulin resistance.^[38,39]^ Additionally, metformin may provide heart and lung benefits to COVID-19 patients by activating AMP-activated protein kinase,^[40]^ and it may inhibit viral invasion through the AMP-activated protein kinase activation and ACE2 phosphorylation pathway.^[41,42]^ Furthermore, Cheng et al reported that taking metformin had no effect on the 30-day mortality rat, but did increase acidos and lactic acidosis, it had no effect on acute respiratory distress syndrome, DIC, acute kidney injury or acute cardiac injury. Metformin decreased the mortality rate of women but not men,^[22]^ and the use of metformin was not related to the incidence or COVID-19-related mortality risk.^[43]^ The results of these studies are contradictory, and the impact of metformin on COVID susceptibility and disease progression remains inconclusive.

Given the massive population of diabetes worldwide and the widespread use of metformin, there is currently a lack of authoritative large-scale research results to guide the choice of hypoglycemic drugs for diabetic patients with COVID-19. The results of several small observational studies are inconsistent, and these observational studies have limitations in methodology, they cannot exclude the interference of confounding factors and reverse causation, which is precisely the advantage of Mendelian randomization studies. We designed a Mendelian randomization study to evaluate the impact of metformin on COVID-19, using Growth differentiation factor 15 (GDF-15), also known as macrophage inhibitory cytokine-1, as a robust biomarker for metformin use.^[25,26]^ We used genetic variations in GDF-15 as exposure and COVID-19 as the outcome and validated COVID-19 in 3 subtypes: susceptibility, hospitalization, and severe illness. Our results showed that metformin had a causal relationship with severe COVID-19 and increased the risk of severe illness in COVID patients. However, metformin had no causal effect on hospitalization or COVID-19 susceptibility in the population. We employed several methods to conduct sensitivity analyses, and the conclusions did not support heterogeneity or pleiotropy. The power calculation was 0.91, indicating the reliability of our results. Additionally, we used the Phenoscanner website tool as a SNP annotator to investigate whether exposed SNPs influenced outcomes through potential risk factors. As exposure instrumental variables, 4 SNPs were identified: rs888663, rs749451, rs1227731, and rs17725099. While rs888663 exhibited a weak correlation with Coronary Artery Disease (5 × 10^−6^), the significant correlation threshold is 5 × 10^−8^. As a Mendelian randomization study, the instrumental variable was strongly correlated with exposure and not correlated with outcome (5 × 10^−8^),by this criterion, it does not violate the conditions of Mendelian randomization, and it is estimated that GDF-15 may not mediate the impact of COVID-19 outcomes through Coronary Artery Disease. Furthermore, rs1227731 increased the granulocyte percentage of myeloid white cells (2.77e−09) and decreased the monocyte percentage of white cells (*P* = 2.10e−08). Reviewing the literature, the biomarkers involved in COVID-19 disease progression include lymphocytes, white blood cells, C-reactive protein, pro-inflammatory cytokines, interleukins (IL)-6, IL-1β, procalcitonin, lactate dehydrogenase, Krebs von den Lungen-6, and ferritin.^[33,44]^ No clear report in the literature links the granulocyte percentage of myeloid white cells to COVID-19 progression or prognosis. Typically, COVID-19 infection triggers changes in the immune system, leading to changes in blood cells; blood cell changes do not cause COVID-19. To verify the stability of the results, we removed rs1227731 and rs888663,we conducted a Mendelian randomization analysis using the same method, we get 4 instrumental variables: rs3746181, rs749451, rs1054565, and rs17725099, none of these SNPs were correlated with COVID-19 or risk factors, and they explained 8.24% of GDF-15 genetic variance (*F* value = 122.1322), rs1054564 was removed because it was palindromic with intermediate allele frequencies, the secondary analysis results are consistent with our results.

Metformin has many contraindications, such as severe renal insufficiency, diseases that may cause tissue hypoxia, severe infections, trauma, liver failure, etc although the indications for metformin have been relaxed, many clinicians still exercise caution when choosing metformin for diabetes patients with COVID-19, which may lead to significant selection bias in observational studies of metformin in patients with COVID-19 and could be one of the reasons for the conflicting conclusions in these studies.

The mechanism by which metformin exacerbates the risk of severe COVID-19 is unclear, the hypoglycemic mechanism of metformin involves lowering hepatic gluconeogenesis, inhibiting the mitochondrial respiratory chain, reducing glucose absorption in the small intestine, and improving insulin sensitivity by increasing glucose uptake and utilization in peripheral tissues,^[45]^ blocking oxidative phosphorylation promotes anaerobic metabolism and increases the risk of lactate accumulation,^[46]^ in severe infection, tissue hypoxia, and anaerobic glycolysis are increased,^[47]^ and some studies suggest that metformin may promote invasion of the SARS-CoV-2 virus and aggravate COVID-19 disease through the AMPK-phosphorylated-ACE2 axis.^[21,48]^

The risk factor analysis shows that metformin may reduce the risk of asthma, obesity, and breast cancer but increase the risk of tuberculosis, some studies suggest that metformin affects asthma by autophagy,^[49]^in contrast, animal experiments suggest that it lowers the affinity between miR-152-3p and DNA methyltransferase 1, downregulates insulin receptor (IR)/insulin-like growth factor 1 receptor level, reduces airway resistance, and has a positive effect on asthma.^[50]^ Metformin affects breast cancer by regulating lncRNAs related to breast cancer^[51]^ and can reduce the risk of breast cancer.^[52]^ The weight-loss effect of metformin is well-known and needs no further elaboration. Further research is needed to clarify the effects and mechanisms of metformin on asthma, breast cancer, tuberculosis, etc.

The statistical effect of IVW was significantly more substantial than MR-Egger,^[53]^ compared to IVW, the MR-Egger model had weaker statistical power, wider confidence intervals, and *P*-values calculated using the same equation as the confidence interval, which explains the non-significant *P*-value observed in our study. Therefore, IVW is usually the primary method, and MR-Egger is only considered when horizontal pleiotropy is present. However, multiple sensitivity analysis methods used in this study did not support the existence of horizontal pleiotropy, indicating that IVW results are more meaningful.^[54]^

Our analysis showed no causal relationship between GDF-15 and diabetes or fasting blood glucose levels because GDF-15 is a use and dosage biomarker for metformin, the relationship between metformin and GDF-15 was not weakened after adjusting for glycated hemoglobin and blood glucose, and it was independent of insulin and proinsulin, these findings suggest that GDF-15 may be a marker of the non-glycemic effects of metformin.^[3,26]^

In conclusion, our results indicate that metformin use increases the risk of severe COVID-19 in diabetic patients but does not affect susceptibility or hospitalization due to COVID-19. We recommend that diabetic patients who have not been infected with COVID-19 can continue to use metformin but should monitor lactate and renal function, for diabetic patients suspected or confirmed to have COVID-19, the use of metformin should be approached with caution. Further research is needed to clarify the mechanisms underlying the association between metformin and COVID-19.

Strengths of our study include being the first to use Mendelian randomization analysis to reveal the association between GDF-15 (a biomarker for metformin) and COVID-19 from a genetic perspective. Compared to observational studies, this approach avoids the influence of confounding factors and reverse causality. However, our study also has some limitations, such as the limited number of instrumental variables exposed and being confined to the European population, which restricts generalizability to other populations.

## Acknowledgments

The authors would like to thank Au Yeung SL et al and IEU, covid-19hg, UKBB, finngen, etc., for open access to the database. We also thank the sponsorship of the Guangxi Health and Wellness Commission Scientific Research Project (approval number: Z20190115).

## Author contributions

**Conceptualization:** Ya Wang.

**Data curation:** Ya Wang.

**Formal analysis:** Ya Wang.

**Funding acquisition:** Ya Wang.

**Investigation:** Ya Wang.

**Methodology:** Ya Wang, Peishan Yao, Kai Li.

**Project administration:** Ya Wang.

**Resources:** Ya Wang.

**Software:** Ya Wang.

**Supervision:** Ya Wang.

**Validation:** Ya Wang.

**Visualization:** Ya Wang.

**Writing – original draft:** Ya Wang.

**Writing – review & editing:** Ya Wang, Peishan Yao, Kai Li, Shanyu Qin.

## Supplementary Material


















