# Supplementary material for: GDF-15 (a biomarker for metformin) and the risk of COVID-19: A two-sample Mendelian randomization study
Source: Medicine (Baltimore). 2023 Sep 29;102(39):e34675. doi: 10.1097/MD.0000000000034675 (PMC10545159; doi:10.1097/MD.0000000000034675)
Supplement: Supplementary file 1 [file medi-102-e34675-s001.docx]

Supplemental Table1 The genetic variations associated with GDF-15 in a study by Au Yeung SL et al. (PMID: 31161347)

| GENE | SNP | Effect_allele | Other_allele | beta | Se | P |
| --- | --- | --- | --- | --- | --- | --- |
| PGPEP1 | rs888663 | T | G | 0.3029 | 0.0244 | 2.64E-35 |
| PGPEP1 | rs3746181 | G | A | 0.3164 | 0.0258 | 1.84E-34 |
| PGPEP1 | rs1363120 | G | C | 0.3147 | 0.0258 | 3.74E-34 |
| PGPEP1 | rs749451 | C | T | 0.2178 | 0.0187 | 2.54E-31 |
| GDF-15 | rs1054564 | C | G | 0.308 | 0.0257 | 3.45E-33 |
| GDF-15 | rs1227731 | A | G | 0.3085 | 0.0257 | 3.37E-33 |
| PGPEP1 | rs3195944 | G | A | 0.3344 | 0.0292 | 2.39E-30 |
| PGPEP1 | rs17725099 | A | G | 0.1346 | 0.0245 | 4.13E-08 |
| GDF-15 | rs16982345 | A | G | 0.1268 | 0.0247 | 2.75E-07 |
