# Supplementary material for: GDF-15 (a biomarker for metformin) and the risk of COVID-19: A two-sample Mendelian randomization study
Source: Medicine (Baltimore). 2023 Sep 29;102(39):e34675. doi: 10.1097/MD.0000000000034675 (PMC10545159; doi:10.1097/MD.0000000000034675)
Supplement: Supplementary file 2 [file medi-102-e34675-s002.docx]

Supplemental Table2,The genetic instruments of exposure of our study

|  | SNP | effect_allele.exposure | other_allele.exposure | beta.exposure | se.exposure | pval.exposure |
| --- | --- | --- | --- | --- | --- | --- |
| 1 | rs888663 | T | G | 0.3029 | 0.0244 | 2.64E-35 |
| 4 | rs749451 | C | T | 0.2178 | 0.0187 | 2.54E-31 |
| 6 | rs1227731 | A | G | 0.3085 | 0.0257 | 3.37E-33 |
| 8 | rs17725099 | A | G | 0.1346 | 0.0245 | 4.13E-08 |
