# Supplementary material for: GDF-15 (a biomarker for metformin) and the risk of COVID-19: A two-sample Mendelian randomization study
Source: Medicine (Baltimore). 2023 Sep 29;102(39):e34675. doi: 10.1097/MD.0000000000034675 (PMC10545159; doi:10.1097/MD.0000000000034675)
Supplement: Supplementary file 4 [file medi-102-e34675-s004.docx]

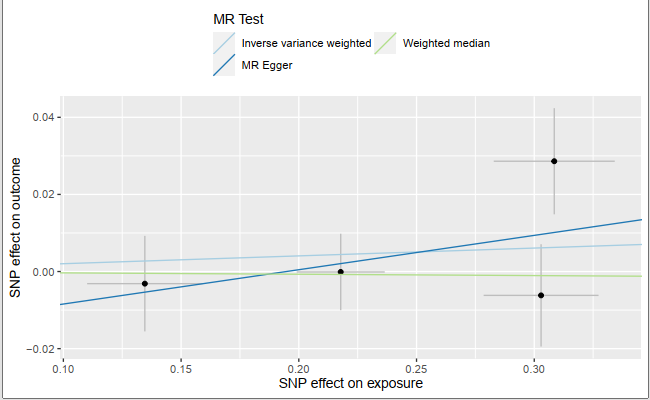


Supplemental Figure 2,scatter plot of GDF-15 and hospitalization of COVID-19, Which reflecting the effect of instrumental variables on COVID-19 hospitalization
